# Association between circulating biomarkers of one-carbon metabolism and glymphatic system function in cognitive decline of Alzheimer’s disease

**DOI:** 10.3389/fneur.2026.1779257

**Published:** 2026-05-11

**Authors:** Yali Chen, Xia Zhou, Kaigui Wang, He Feng, Bo Tian, Yating Tang, Xiaoqun Zhu, Zhongwu Sun

**Affiliations:** Department of Neurology, The First Affiliated Hospital of Anhui Medical University, Hefei, Anhui, China

**Keywords:** Alzheimer’s disease, cognition, DTI-ALPS, folate, glymphatic function, homocysteine, one-carbon

## Abstract

**Introduction:**

Dysfunction in both one-carbon metabolism (OCM) and the glymphatic system has been implicated in the pathogenesis of Alzheimer’s disease (AD). However, the potential interrelationship remains poorly understood. This study aimed to investigate the association between OCM biomarkers and glymphatic function in AD and explore their combined effects on cognition.

**Methods:**

A total of 210 participants were enrolled, including 68 with normal cognition (NC), 75 with mild cognitive impairment due to AD (AD-MCI), and 67 with dementia due to AD (AD-D). Circulating OCM biomarkers were measured, including serum folate, vitamin B12, and homocysteine levels. Glymphatic-related function was evaluated using the diffusion tensor imaging-analysis along the perivascular space (DTI-ALPS) index, and comprehensive neuropsychological assessments were performed.

**Results:**

Serum folate levels (*p* = 0.020) and DTI-ALPS index (*p* < 0.001) differed significantly between NC and AD groups, with more pronounced differences among female (*p* = 0.005; 0.001) and late-life (*p* = 0.046; 0.021) subgroups. In AD patients, folate levels were positively correlated with DTI-ALPS index (*r* = 0.212, p_FDR_ = 0.032), and both were associated with domain-specific cognitive performance. The low-risk group (high folate and high DTI-ALPS index) outperformed the high-risk group in memory (*p* = 0.001) and processing speed (*p* = 0.005), and showed higher MMSE (*p* = 0.021) and memory scores (*p* = 0.003) than the moderate-risk group.

**Conclusion:**

Lower serum folate levels and reduced DTI-ALPS index were associated with poorer cognitive performance in AD. Their co-occurrence was linked to worse cognitive outcomes. These findings suggest a potential association between metabolic status and glymphatic function in AD, which requires confirmation in longitudinal studies.

## 1 Introduction

Alzheimer’s disease (AD) is a chronic, progressive, and irreversible neurodegenerative disorder of the central nervous system. It is currently the most common cause of dementia globally and has a substantial financial impact (1). The latest epidemiological data indicate that between 1992 and 2021, the number of AD cases worldwide increased from 4.078 million to 9.837 million, and is expected to surpass 19 million by 2036 (2). The formation of neurofibrillary tangles composed of hyperphosphorylated tau protein and the aberrant deposition of amyloid-β (Aβ) plaques are the two main pathogenic hallmarks of AD. These abnormal changes trigger a series of events that eventually result in synaptic dysfunction, neuronal damage, and progressive neurodegeneration (3). However, it is important to highlight that the pathophysiological process of AD is thought to begin silently approximately 20 years before dementia is diagnosed clinically. Thus, this prolonged “preclinical” window offers a valuable and short-lived strategic chance for intervention and therapy (4). Serum nutritional biomarkers such as folate, along with metabolism-related factors, have been implicated in early intervention for AD, including one-carbon metabolism (OCM) and the brain’s glymphatic clearance function (5, 6).

OCM is formed by the synergistic action of the folate and the methionine cycle, whose sustained operation depends on adequate folate and vitamin B12. 5-Methylenetetrahydrofolate (5-MTHF) provides methyl groups for vitamin B12-dependent methionine synthase to convert homocysteine (Hcy) into methionine. Alternatively, Hcy can enter the transsulfuration pathway catalyzed by cystathionine β-synthase (CBS) and vitamin B6 to generate cysteine. The first process generates S-adenosylmethionine (SAM) (7), which supplies methyl groups for DNA, histone, and protein methylation. OCM is essential for maintaining genomic stability, epigenetic regulation, and the synthesis of membrane phospholipids required for synaptic plasticity (8). As the central hub of cellular methylation and redox metabolism, the OCM plays a vital role in maintaining homeostasis within the nervous system. A growing body of research links OCM to the onset and progression of AD (9). Folate is a water-soluble B vitamin obtained from dietary sources and serves as both a carrier and donor of one-carbon units (10). By providing methyl groups (via SAM), it participates in numerous bodily reactions such as monoamine neurotransmitter synthesis (11), myelin formation (12), and the promotion of lifelong neural cell growth and repair (13). Multiple independent population studies consistently reported that serum folate levels in AD patients are lower than those in healthy controls (14, 15). However, folate deficiency damages DNA and mitochondrial DNA (mtDNA), leading to oxidative stress and excessive production of reactive oxygen species (ROS). This cascade is recognized as an early-stage factor in the pathogenesis of AD (16), ultimately leading to the damage and death of neurons in AD-related brain regions (17). Singh et al. suggested that folate supplementation is believed to exert antioxidant and memory-enhancing effects (18), preventing β-amyloid-induced oxidative stress and neurotoxicity (19). Corrada et al. and Luchsinger et al. found that increased dietary and supplemental folate intake may reduce the risk of AD (20, 21). Vitamin B12 is an essential water-soluble B-complex nutrient derived from dietary sources (22). It serves as a cofactor for methyl transfer in the OCM and exhibits strong synergistic effects with folate in OCM and nervous system maintenance. It can suppress inflammation-mediated oxidative damage by regulating the expression of cytokines and neurotrophic factors. However, neuroinflammation is a core driver in the pathological progression of AD (23, 24). Inadequate dietary folate and B-vitamin intake, mutations in methylenetetrahydrofolate reductase (MTHFR), and impaired transsulfuration are thought to raise Hcy levels in AD patients. MTHFR catalyzes the reduction of 5,10-MTHF to 5-MTHF, thereby furnishing the methyl donor for Hcy remethylation (9). Hyperhomocysteinemia-induced oxidative stress and neurotoxicity have been identified as factors associated with AD (25). Various clinical trials have indicated that folate or combined B-vitamin supplementation, by lowering Hcy levels, can slow cognitive decline and reduce the risk of dementia (26, 27). Disruption of the OCM activates a central inflammatory cascade and exacerbates oxidative stress, inducing structural damage to neuronal proteins and generating multiple abnormal metabolic byproducts, thereby accumulating toxic waste within brain tissue (8). In summary, impairment of the OCM pathway leads to significant neuronal damage and toxicity.

The discovery of the glymphatic system has fundamentally revolutionized our understanding of brain waste clearance mechanisms (55). Its core function is the convective exchange between cerebrospinal fluid (CSF) and interstitial fluid (ISF), aimed at clearing neurotoxic metabolic wastes such as Aβ and tau proteins (56). In 2012, Nedergaard’s team utilized two-photon microscopy to demonstrate for the first time in a mouse model that CSF flows into the brain parenchyma along periarterial spaces, exchanges with ISF via convection, and ultimately drains along perivenous spaces carrying metabolic waste. This seminal work introduced the concept of the “glymphatic system” (57). In 2013, the same team further elucidated the critical role of aquaporin-4 (AQP4) on astrocytic endfeet in driving CSF influx, showing that AQP4 deletion or depolarization significantly impairs clearance efficiency (58). Alterations in AQP4 expression and polarization in brain tissue from patients with AD have been confirmed to be closely associated with disease progression (59). Studies in 2014 further revealed that glymphatic function is markedly enhanced during non-rapid eye movement sleep: during deep sleep, the interstitial space expands by approximately 60%, reducing flow resistance and increasing clearance efficiency, while elevated norepinephrine levels during wakefulness constrict the interstitial space, inhibiting this function (60). Vascular pulsation, particularly arterial pulsation, serves as another key driving force for CSF flow, with respiratory rhythm synergistically enhancing clearance efficiency (61). In AD, the capacity of the glymphatic system to clear Aβ and tau proteins is impaired, leading to their pathological accumulation (62). A growing body of evidence indicates a significant association between the pathological progression of AD and reduced efficiency of metabolic waste clearance from the brain. Clinical imaging studies have shown that glymphatic function indicators in AD patients, such as the DTI-ALPS index, are significantly reduced and closely correlate with cognitive decline (63). Given its critical role in brain waste clearance, the glymphatic system has emerged as a promising diagnostic and therapeutic target for neurological disorders. However, to date, no studies have explored the interaction between circulating biomarkers of the OCM and the brain’s glymphatic waste clearance system in AD patients, nor their impact on cognitive function. In this study, we compared the DTI-ALPS index among patients with varying degrees of cognitive impairment with differences in circulating biomarkers of the OCM. We further analyzed the association patterns between the DTI-ALPS index and related serum biomarkers with scores across various cognitive domains. The findings may shed light on the potential linking of OCM pathways to AD-related cognitive impairment, and provide evidence-based support for developing personalized intervention strategies based on nutritional regulation.

## 2 Methods

### 2.1 Participants

This study enrolled 210 right-handed Han Chinese participants aged 45–80 years from the First Affiliated Hospital of Anhui Medical University, comprising 67 patients with Alzheimer’s disease dementia (AD-D), 75 patients with mild cognitive impairment due to AD (AD-MCI), and 68 cognitively normal controls (NC). All participants underwent neuropsychological assessment, laboratory analyses, and magnetic resonance imaging (MRI) examination. This study was approved by the Institutional Ethics Committee of the hospital and conducted in accordance with the principles of the Declaration of Helsinki (approval number: Quick-PJ 2023-13-62). All participants or their legally authorized representatives received detailed information about the study and provided written informed consent. The main demographic and clinical characteristics of the study participants are presented in Table 1. Clinical diagnoses of AD-D patients met the National Institute on Aging and Alzheimer’s Association (NIA-AA) (28) criteria: (1) Met criteria for probable AD; (2) Mini-Mental State Examination (MMSE) score ≤ 24 (29); (3) Clinical Dementia Rating (CDR) score ≥ 1. Patients with AD-MCI were also clinically diagnosed according to the NIA-AA criteria, with the following diagnostic outcomes (30): (1) Subjective complaints of cognitive impairment by the individual, informants, or clinicians; (2) Significant deficits in memory or other cognitive domains (1.5 standard deviations below age-and education-adjusted norms), but without significant impairment in daily functioning; (3) CDR score of 0.5. Inclusion criteria for the NC group were as follows: (1) Absence of memory loss symptoms; (2) MMSE score > 26; (3) CDR score of 0. Most cases of mild cognitive impairment and dementia due to AD were diagnosed according to the 2011 NIA-AA diagnostic criteria, with some patients included according to the 2018 ATN diagnostic framework (31). Participants were excluded if any of the following were present: (1) Cognitive impairment potentially caused by other diseases, such as Parkinson’s disease dementia, neurosyphilis, HIV infection, or thyroid dysfunction; (2) Use of folate or other B-complex vitamins, nootropics, methotrexate, isoniazid, carbamazepine, or levodopa within the previous 6 months; (3) Individuals with a history of severe anxiety, depression, somatic disorders, mental illness, or electroconvulsive therapy; (4) Systemic diseases, including severe liver or kidney disease, tumors, autoimmune disorders, or other serious digestive system diseases; (5) Severe dementia, poor compliance, presence of MRI contraindications, or refusal to provide informed consent.

**Table 1:** Demographic data and cognition.

Characteristics	NC (*n* = 68)	AD-MCI (*n* = 75)	AD-D (*n* = 67)	*p* value
Age, (years)	59.5 (56.25, 66.50)	64 (57.00, 71.00)	63 (58.00, 70.00)	0.150
Sex, *n* (%)				0.678
Female	43 (63.2%)	42 (56.0%)	40 (59.7%)	
Male	25 (36.8%)	33 (44.0%)	27 (40.3%)	
Education (years)	8.5 (6, 12)	9 (6, 12)	8 (3, 10)	0.072
BMI, kg/m^2^	23.38 (21.53, 24.71)	23.05 (21.09, 25.35)	22.86 (20.34, 24.00)	0.076
Vascular risk factors				
Hypertension, *n* (%)	19 (27.9%)	27 (38.0%)	21 (31.8%)	0.441
Diabetes, *n* (%)	4 (5.9%)	11 (14.7%)	5 (7.5%)	0.159
Hyperlipidemia, *n* (%)	16 (23.5%)	13 (17.3%)	8 (11.9%)	0.209
Smoking, *n* (%)	11 (16.2%)	14 (18.7%)	17 (25.4%)	0.384
Drinking, *n* (%)	15 (22.1%)	18 (24.0%)	22 (32.8%)	0.314
APOE gene				**<0.001**
ε4 carriers, *n* (%)	9 (14.3%)	21 (28.8%)	29 (45.3%)	
ε4 non-carriers, *n* (%)	54 (85.7%)	52 (71.2%)	35 (54.7%)	
Cognitive function				
MMSE	0.51 (−0.49, 0.51)	−2.49 (−6.49, −0.49)	−13.49 (−17.49, −10.49)	**<0.001**
MoCA	0.00 ± 1.00	−1.42 ± 1.61	−5.10 ± 1.55	**<0.001**
Memory	0.15 (−2.49, 0.62)	−1.36 (−2.09, −0.15)	−2.49 (−2.96, −1.96)	**<0.001**
Executive functions	0.10 (−0.26, 0.57)	−0.28 (−0.86, 0.32)	−0.95 (−3.34, −0.18)	**0.003**
Attention	0.14 (−0.55, 0.513780)	−0.02 (−0.84, 0.64)	−1.30 (−2.02, −0.68)	**<0.001**
Processing speed	0.00 (−0.51, 0.49)	−0.08 (−0.81, 0.26)	−1.86 (−2.83, −0.85)	**<0.001**
Visuospatial abilities	0.22 (−0.86, 0.75)	0.32 (−0.86, 0.75)	−3.00 (−4.07, −1.39)	**<0.001**

Continuous variables were assessed for intergroup differences using one-way ANOVA or Kruskal-Wallis tests. Categorical variables were compared using χ^2^ tests. All cognitive measures are presented as Z-scores.

NC, normal control; AD-MCI, mild cognitive impairment due to Alzheimer’s disease; AD-D, Alzheimer’s disease dementia; MMSE, Mini-Mental State Examination; MoCA, Montreal Cognitive Assessment.

Bold values indicate *p* < 0.05 (statistically significant).

### 2.2 Neuropsychological assessments

A battery of 10 tasks was administered by two licensed neuropsychologists who were blinded to participants’ group assignments. Global cognitive functions were assessed using the Mini-Mental State Examination (MMSE) and the Montreal Cognitive Assessment (MoCA). Domain-specific assessment tools were as follows (64–66): memory: Auditory Verbal Learning Test (AVLT), which evaluates verbal learning and delayed recall; executive function: Stroop Color-Word Test C (SCWT-C), which assesses the ability to identify color words under interference conditions; Trail Making Test B (TMT-B), used to evaluate executive function; and Verbal Fluency Test (VFT), which examines language proficiency; attention: Digit Span Test (DST), which measures working memory and attention; visuospatial skills: Clock Drawing Test (CDT), which assesses visuoconstructional skills (67); processing speed: Trail Making Test A (TMT-A), evaluating executive function; and Stroop Test A and B (SCWT-A, SCWT-B), which assess color dot identification and color-word identification, respectively; functional status: Activities of Daily Living Scale (ADL), which measures the ability to perform basic activities necessary for daily living; and Clinical Dementia Rating Scale (CDR), used for the diagnosis of dementia and assessment of disease severity.

Raw cognitive scores of the AD group were converted to Z-scores using the mean and standard deviation of the control group (68). Z-scores for time-dependent tests (TMT-A/B, SCWT-A/B/C) were reversed to negative values to ensure that higher scores consistently represented better performance. Missing data were handled using listwise deletion: participants with missing data for any test within a domain were excluded from that domain’s composite analysis. Domain composite scores were calculated as the mean of constituent Z-scores within each cognitive domain. The internal consistency of each cognitive domain composite score in the study is presented in Supplementary Table S1.

### 2.3 Biochemical and genetic analyses

After an overnight fast of 12–14 h, venous blood was drawn from the antecubital vein in the morning. Three milliliters of whole blood were collected into standard biochemical tubes, allowed to clot at 4 °C for 30 min, then centrifuged at 1,500 rpm for 10 min; the supernatant was collected. Serum Hcy was measured using a chemiluminescent immunoassay. Folate and vitamin B12 concentrations were determined via an electrochemiluminescent immunoassay platform.

For APOE genotyping, 2 mL blood was collected into an EDTA anticoagulant tube, centrifuged at 3,500 rpm for 8 min, and 500 μL of whole blood was transferred to EP tubes for storage at −80 °C. Genomic DNA was isolated from whole-blood aliquots using the Aidlab DN01 rapid-extraction kit. The APOE polymorphic sites rs429358 and rs7412 were analyzed by BGI using PCR amplification, electrophoresis, amplification fragment purification, and SNP analysis.

### 2.4 MRI acquisition

Whole-brain imaging was performed using a 24-channel phased-array head coil on a GE Healthcare 3.0 T MRI system (Discovery MR750w, Milwaukee, Wisconsin, United States). Subjects were instructed to lie supine with eyes closed, remain motionless, and avoid falling asleep or engaging in active thought. Two sequences were acquired: (1) High-resolution three-dimensional T1-weighted sequence (3D-bravo T1WI): TR/TE 8.46/3.25 ms, flip angle 12°, field of view 256 × 256 mm^2^, matrix 256 × 256, 188 sagittal slices, 1 mm isotropic slice thickness, scan duration 4 min 56 s; (2) Diffusion Tensor Imaging (DTI): TR/TE 10,000/74 ms, flip angle 90°, field of view 256 × 256 mm^2^, matrix 128 × 128, 50 axial slices, slice thickness 3 mm, no gap, 64 directions (b = 1,000 s mm^2^) plus 5 b = 0 images, total acquisition time 11 min 40 s.

### 2.5 DTI data processing

**Figure 1:**
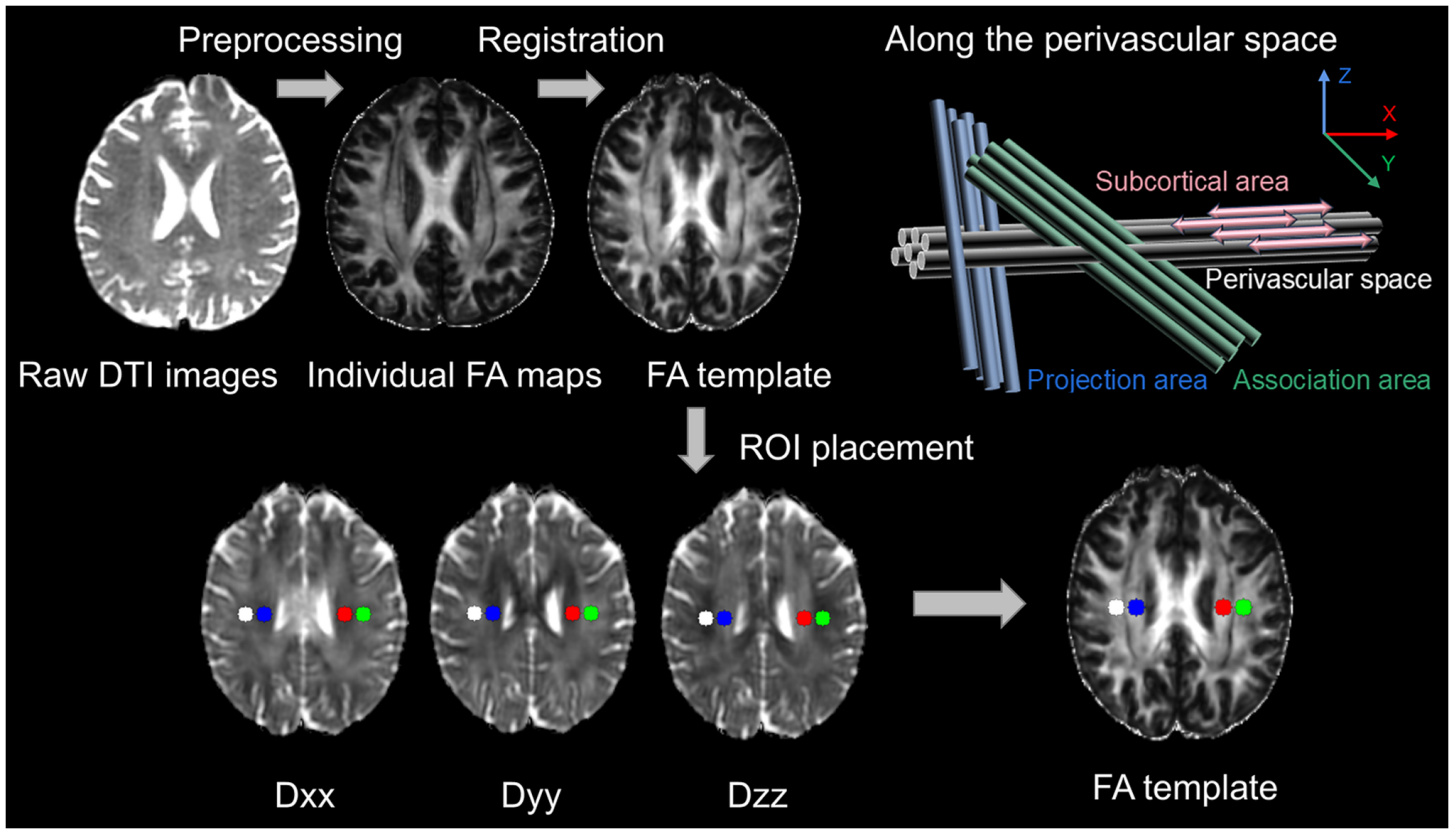
MRI workflow schematic of the DTI-ALPS index. Raw DTI images were preprocessed to generate fractional anisotropy (FA) maps. Each subject’s FA map was registered to the FA template. Four spherical ROIs of 5 mm diameter were placed at the centers of the projection and association fiber systems. The diffusion coefficients along the *x*, *y*, and *z* axes were measured for each ROI, and the bilateral DTI-ALPS indices were calculated.

The ALPS index is calculated from DTI using a semi-automated and highly reliable pipeline developed and validated by Taoka et al. (69). Following visual inspection by two neuroradiologists to exclude structural abnormalities and severe artifacts, raw DTI data were denoised using MP-PCA and corrected for Gibbs ringing artifacts in MRtrix3 (v3.0.3). The FSL (v6.0.5) topup-eddy pipeline was employed for distortion, eddy current, and motion correction, with *b* = 0 images acquired in both anterior–posterior (AP) and posterior–anterior (PA) phase-encoding directions to leverage their complementary distortion patterns for correcting geometric distortions caused by B0 field inhomogeneity (70, 71). For motion quality control, framewise displacement (FD) was used to quantify head motion, with frames exhibiting FD > 1.5 mm or rotation > 2° flagged as high-motion frames. The eddy command automatically replaced volumes affected by >2 mm translation or >2° rotation. Participants were excluded if high-motion frames exceeded 15% or mean FD > 1.5 mm (72, 73). The FSL dtifit tool was used to generate Dxx, Dyy, Dzz, and FA maps. FA maps were linearly (FLIRT) and nonlinearly (FNIRT) registered to the JHU-ICBM-FA-1 mm template; participants with poor registration identified through visual quality inspection were excluded. Transformation matrices were applied to all diffusion maps using trilinear interpolation. In template space, 5 mm spherical ROIs were defined at MNI coordinates corresponding to the superior corona radiata (SCR) and superior longitudinal fasciculus (SLF), and applied to all subjects’ diffusion maps (Figure 1) (74). The DTI-ALPS index is defined as the mean of bilateral DTI-ALPS indices. The unilateral DTI-ALPS index is calculated by summing the x-axis diffusion value (Dxproj) of the SCR in the lateral projection fiber region (Dxproj) with the x-axis diffusion value (Dxassoc) of the associative fiber region SLF, divided by the sum of the y-axis diffusion value (Dyproj) of the projection fiber region SCR and the z-axis diffusion value (Dzassoc) of the associative fiber region SLF. The calculation formula is as follows:


$$\mathrm{ALPS index}=\frac{\mathrm{mean}\;\left(\mathrm{Dxproj,}\;\;\mathrm{Dxassoc}\right)}{\mathrm{mean}\;\left(\mathrm{Dyproj,}\;\;\mathrm{Dzassoc}\right)}$$

### 2.6 Statistical analysis

All statistical analyses were performed using R version 4.4.1,^1^ and *p* < 0.05 was considered statistically significant. Categorical variables were presented as counts (percentages) and were compared using the χ^2^ test. Continuous variables were described as mean ± standard deviation (mean ± SD) when normally distributed, and as median (interquartile range) [M (Q25, Q75)] when non-normally distributed. Normality was assessed by the Shapiro–Wilk test. One-way ANOVA or the Kruskal-Wallis H test was employed to evaluate differences among the NC, AD-MCI, and AD-D groups, with *post-hoc* pairwise comparisons corrected by the Bonferroni method. To examine whether the observed associations differed across subpopulations, we performed subgroup analyses stratified by age (<60 vs. ≥60 years), sex (male vs. female), and clinical status (NC vs. AD group). Between-group comparisons were performed using the independent-samples *t*-test or the Mann–Whitney U test. Associations between the DTI-ALPS index and serum concentrations of folate, vitamin B12, and Hcy were assessed with Pearson’s or Spearman’s correlation, and the resulting *p*-values were adjusted using the false discovery rate (FDR) procedure. Partial correlation analyses were conducted to evaluate the relationships of serum folate, vitamin B12, Hcy, and the whole-brain and bilateral ALPS indices with cognitive performance, while adjusting for age, sex, years of education, and APOE *ε* 4 status, with FDR correction applied again. Multiple linear regression was used to investigate the combined effects of folate status and glymphatic function on cognition, with adjustment for age, sex, education level, and APOE ε4 status.

## 3 Results

### 3.1 Demographic and cognitive variables

A total of 210 subjects were enrolled, comprising 67 cases in the Alzheimer’s disease-related dementia (AD-D) group (27 males, 40 females), 75 participants in the Alzheimer’s disease-related mild cognitive impairment (AD-MCI) group (33 males, 42 females), and 68 cases in the cognitively normal control (NC) group (25 males, 43 females). Demographic and cognitive data for participants are summarized in Table 1. No significant differences were observed among the three groups in age, gender, years of education, body mass index (BMI), or vascular risk factors such as smoking, alcohol consumption, hypertension, diabetes, and hyperlipidemia (all *p* > 0.05). The proportion of APOE ε4 carriers was significantly greater in the AD-D group than in both the NC and AD-MCI groups (*p* < 0.05). Compared with the NC group, both the AD-D and AD-MCI groups performed significantly worse on all neuropsychological tests (*p* < 0.001).

### 3.2 Differences in the DTI-ALPS index among the NC, AD-MCI, and AD-D groups

**Figure 2:**
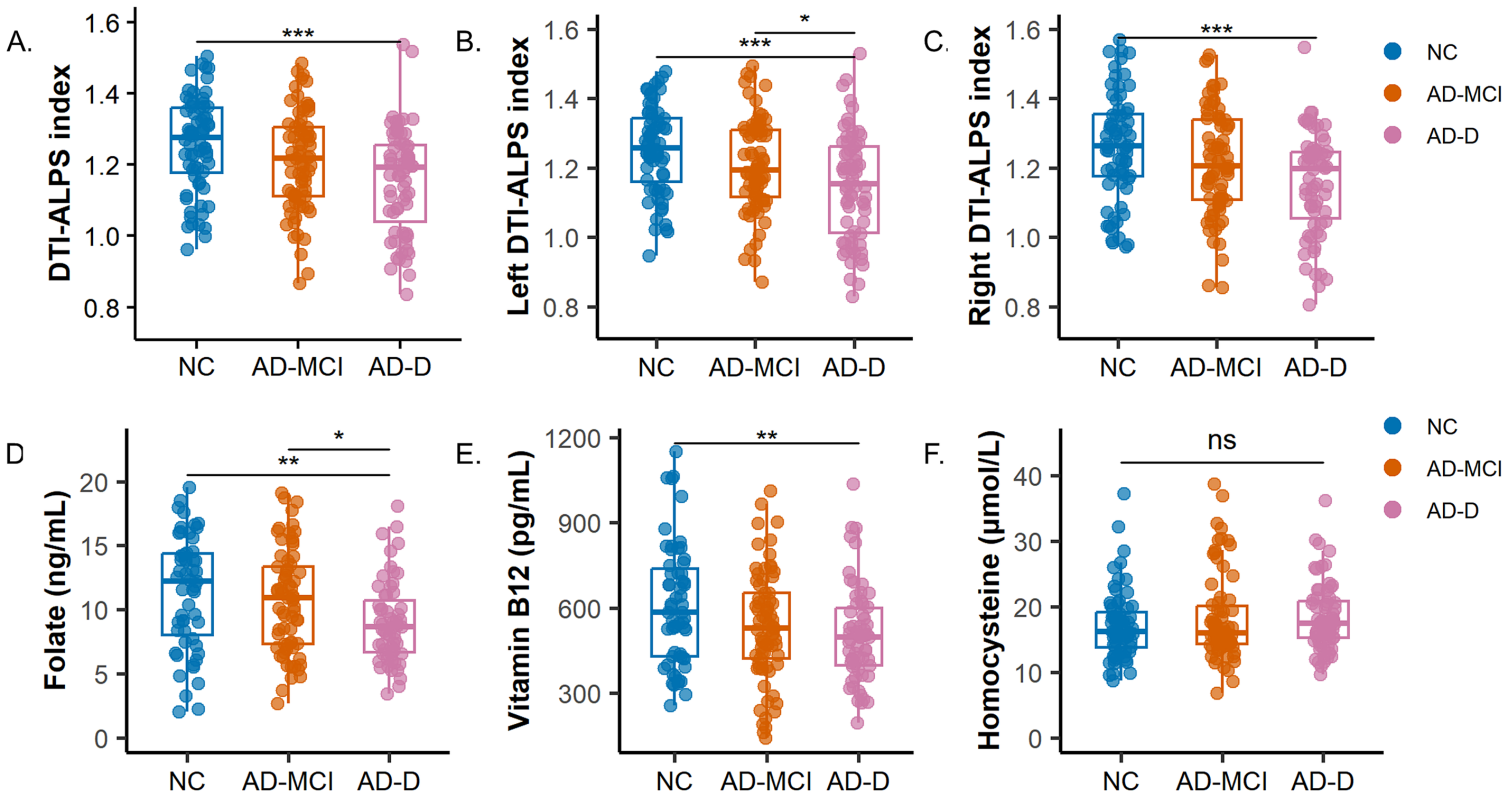
Box plots illustrate differences in the DTI-ALPS index and circulating biomarkers of one-carbon metabolism among NC, AD-MCI, and AD-D groups **(A–C)** DTI-ALPS index. **(D)** Serum folate levels. **(E)** Serum vitamin B12 levels. **(F)** Serum Hcy levels. *p < 0.05; **p < 0.01; ***p < 0.001.

The DTI-ALPS index exhibited a graded decrease across groups. Significant differences were observed in the whole brain DTI-ALPS index (*p* < 0.001), as well as in the left (*p* < 0.001) and the right indices (*p* = 0.001). *Post-hoc* analysis revealed that patients in the AD-D group exhibited significantly lower overall DTI-ALPS index (1.16 ± 0.15 vs. 1.26 ± 0.13, *p* < 0.001), left DTI-ALPS index (1.15 ± 0.15 vs. 1.25 ± 0.13, *p* < 0.001), and right DTI-ALPS index (1.16 ± 0.16 vs. 1.26 ± 0.15, *p* < 0.001) than those in the NC group. The left DTI-ALPS index in the AD-D group was also significantly lower than that in the AD-MCI group (1.15 ± 0.15 vs. 1.21 ± 0.13, *p* = 0.043), while no statistically significant difference was observed between the AD-MCI and NC groups (Table 2; Figures 2A–C).

**Table 2:** Circulating biomarkers of one-carbon metabolism and DTI-ALPS index.

Characteristics	NC (*n* = 68)	MCI (*n* = 75)	AD (*n* = 67)	*p* value^a^	*p* value^b^
DTI-ALPS					
Left DTI-ALPS index	1.25 ± 0.13	1.21 ± 0.13	1.15 ± 0.15	**<0.001**	**<0.001** ^c^
Right DTI-ALPS index	1.26 ± 0.15	1.22 ± 0.15	1.16 ± 0.16	**0.001**	**<0.001** ^c^
DTI-ALPS index	1.26 ± 0.13	1.21 ± 0.14	1.16 ± 0.15	**<0.001**	**<0.001** ^c^
Serological markers					
Folate, ng/mL	12.22 (7.73, 14.45)	10.92 (7.29, 13.44)	8.66 (6.57, 11.14)	**0.004**	**0.004** ^c^
Vitamin B12, pg./mL	595.5 (430.10, 757.50)	528 (413.50, 653.50)	497.5 (396.50, 606.50)	**0.012**	**0.010** ^c^
Homocysteine, μmol/L	16.20 (13.61, 19.56)	16.07 (14.31, 20.69)	17.52 (15.18, 21.09)	0.339	

DTI-ALPS, diffusion tensor image analysis along the perivascular space.

a *p* value for comparison among NC, AD-MCI, and AD-D by ANOVA or Kruskal–Wallis H tests.

b Only listed *p* < 0.05 for multiple comparisons.

c NC vs AD-D.

Bold values indicate *p* < 0.05 (statistically significant).

### 3.3 Differences in the circulating biomarkers of one-carbon metabolism among the NC, AD-MCI, and AD-D groups

Significant group differences were observed in serum folate (*p* = 0.004) and vitamin B12 levels (*p* = 0.012). *Post hoc* comparisons indicated that AD-D patients had lower folate levels (*p* = 0.004) and vitamin B12 levels (*p* = 0.010) compared to the NC group. The AD-D group had lower folate levels (*p* = 0.049) than the AD-MCI group, while no significant difference was found between the AD-MCI and NC groups. No differences were observed in Hcy levels across the groups (Table 2; Figures 2D–F).

### 3.4 Stratified analysis

**Figure 3:**
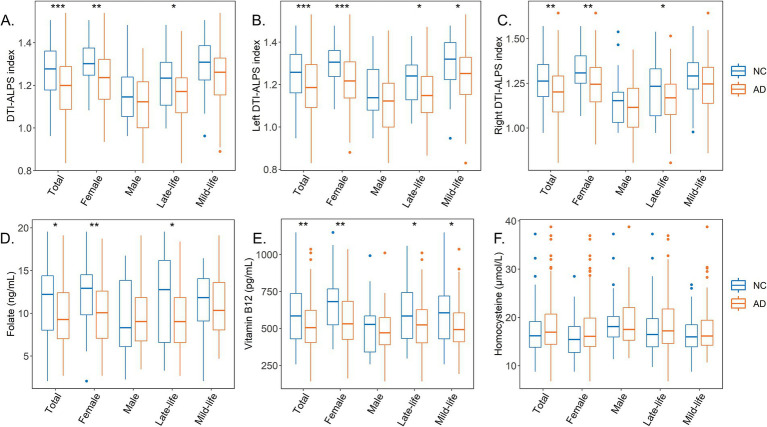
Box plots illustrate differences in circulating biomarkers of one-carbon metabolism and DTI-ALPS index between the NC and AD groups in the stratified analysis. **(A)** DTI-ALPS index. **(B)** Left DTI-ALPS index. **(C)** Right DTI-ALPS index. **(D)** Serum folate levels. **(E)** Serum vitamin B12 levels. **(F)** Serum Hcy levels. *p < 0.05; **p < 0.01; ***p < 0.001.

In the AD group (including both AD-D and AD-MCI), participants exhibited significantly lower serum folate and vitamin B12 levels, along with reduced whole-brain and bilateral ALPS indices compared to the NC group. Similar results were observed in the female and late-life subgroups (Figure 3; Supplementary Table S2).

### 3.5 Association between circulating biomarkers of one-carbon metabolism and DTI-ALPS index

**Figure 4:**
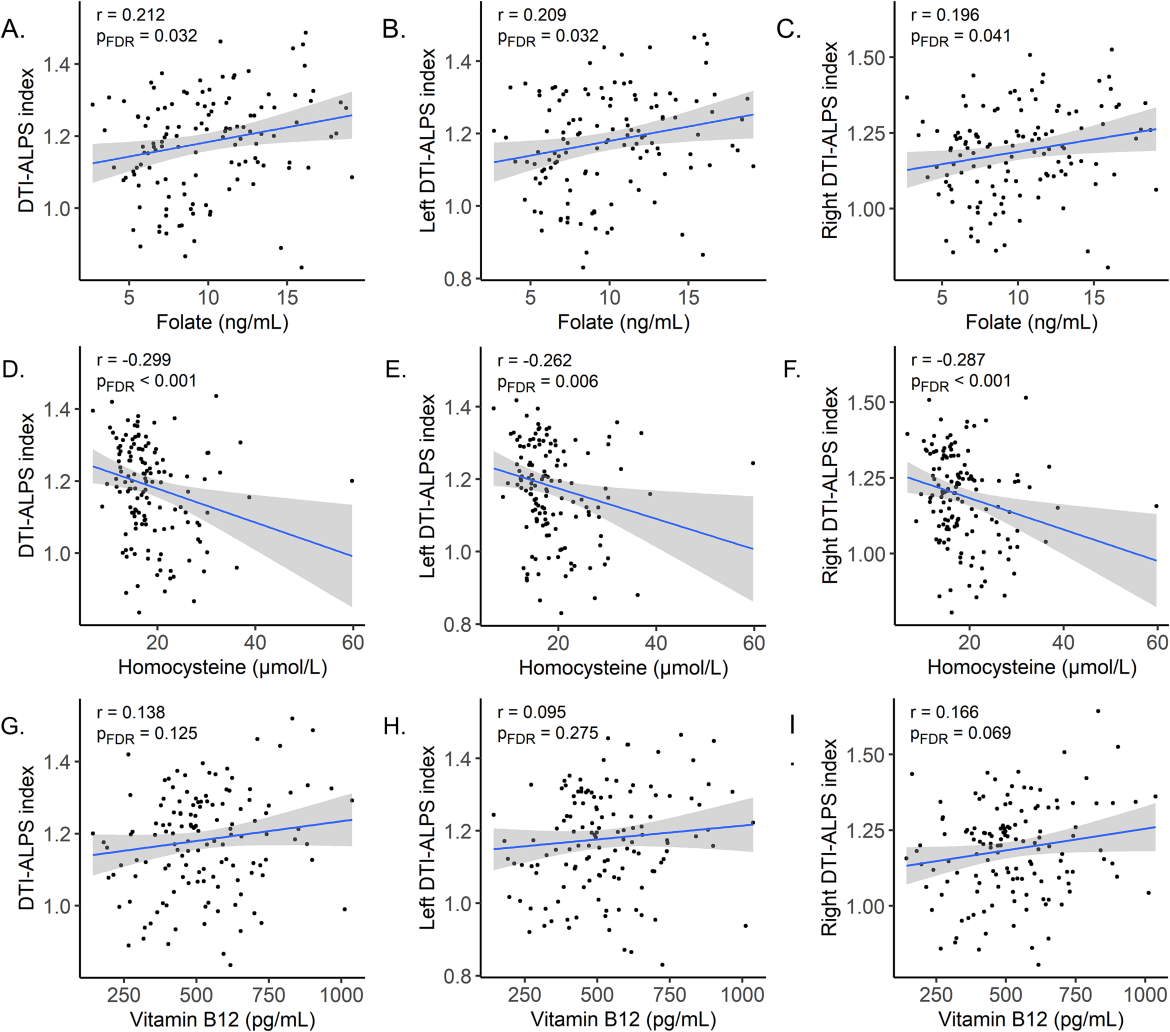
Scatter plots were used to examine the correlations between circulating one-carbon cycle biomarkers and the DTI-ALPS index. **(A)** Folate versus DTI-ALPS index. **(B)** Folate versus left DTI-ALPS index. **(C)** Folate versus right DTI-ALPS index. **(D)** Hcy versus DTI-ALPS index. **(E)** Hcy versus left DTI-ALPS index. **(F)** Hcy versus right DTI-ALPS index. **(G)** Vitamin B12 versus DTI-ALPS index. **(H)** Vitamin B12 versus left DTI-ALPS index. **(I)** Vitamin B12 versus right DTI-ALPS index.

As shown in Figure 4, in the AD group, the serum folate levels were positively correlated with the overall DTI-ALPS index of the whole brain (*r* = 0.212, *p*_FDR_ = 0.032), the left DTI-ALPS index (*r* = 0.209, *p*_FDR_ = 0.032), and the right DTI-ALPS index (*r* = 0.196, *p*_FDR_ = 0.041). In contrast, serum Hcy levels were negatively correlated with the overall DTI-ALPS index of the whole brain (*r* = −0.299, *p*_FDR_ < 0.001), the left DTI-ALPS index (*r* = −0.262, *p*_FDR_ = 0.006), and the right DTI-ALPS index (*r* = −0.287, *p*_FDR_ < 0.001). No significant associations were observed between serum vitamin B12 concentrations and any DTI-ALPS measure. Detailed results are presented in Supplementary Table S3.

### 3.6 The relationship between the DTI-ALPS index and circulating biomarkers of one-carbon metabolism with cognitive function

**Figure 5:**
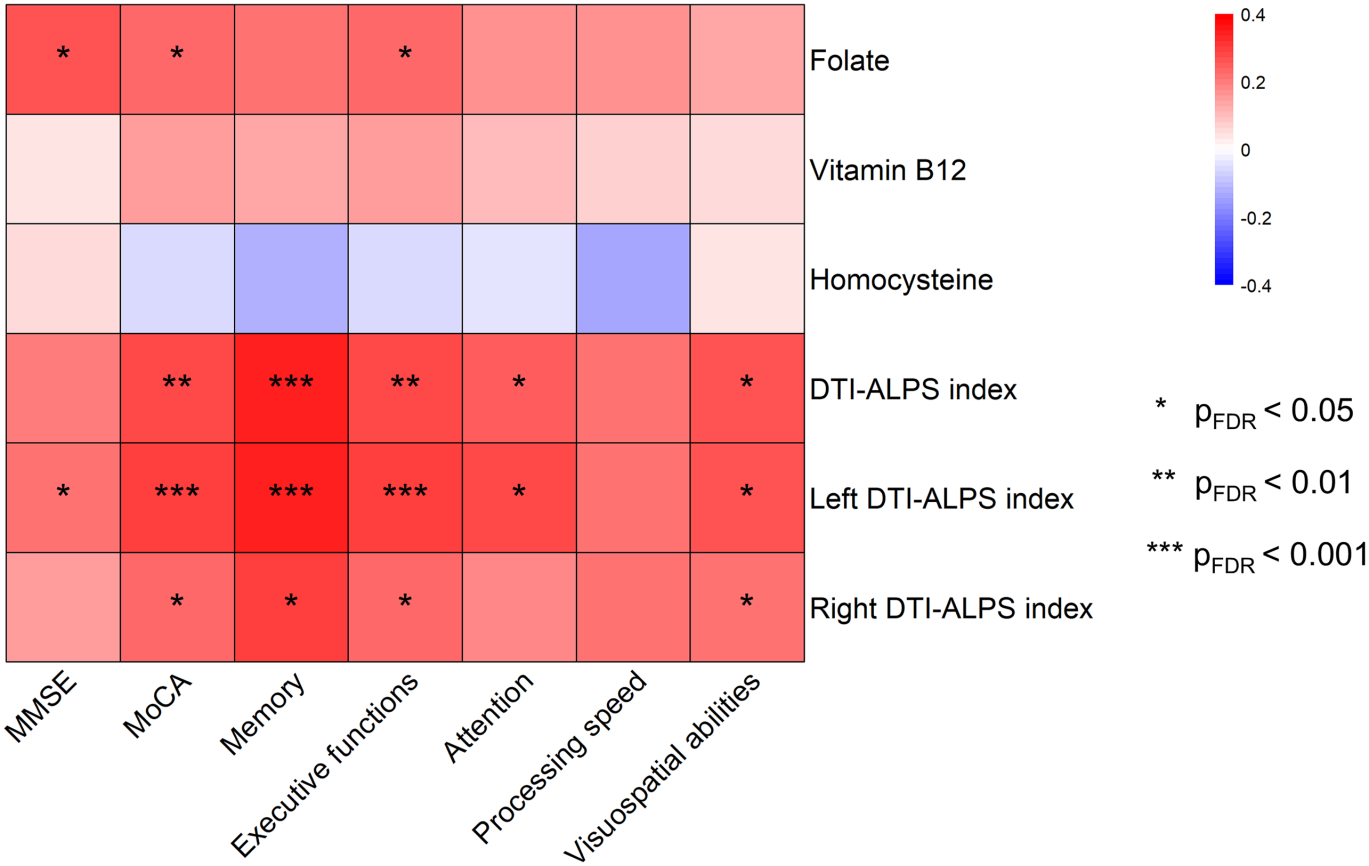
The heatmap illustrates the respective associations of the DTI-ALPS index and circulating one-carbon metabolism biomarkers with cognitive function. Serum folate, along with the whole-brain and bilateral ALPS indices, showed significant positive correlations with some cognitive scores, whereas vitamin B12 and Hcy did not.

As illustrated in Figure 5, serum folate was positively associated with MMSE (*r* = 0.258, *p*_FDR_ = 0.019), MoCA (*r* = 0.238, *p*_FDR_ = 0.030) and executive function (*r* = 0.238, *p*_FDR_ = 0.030), after adjusting for age, gender, education, and APOE ε4 status. The whole-brain DTI-ALPS index correlated positively with MoCA (*r* = 0.282, *p*_FDR_ = 0.007), memory (*r* = 0.339, *p*_FDR_ < 0.001), executive function (*r* = 0.283, *p*_FDR_ = 0.007), attention (*r* = 0.245, *p*_FDR_ = 0.025) and visuospatial abilities (*r* = 0.255, *p*_FDR_ = 0.027). Similarly, the left DTI-ALPS index showed positive associations with MMSE (*r* = 0.221, *p*_FDR_ = 0.030), MoCA (*r* = 0.299, *p*_FDR_ < 0.001), memory (*r* = 0.343, *p*_FDR_ < 0.001), executive function (*r* = 0.299, *p*_FDR_ < 0.001), attention (*r* = 0.279, *p*_FDR_ = 0.011) and visuospatial abilities (*r* = 0.255, *p*_FDR_ = 0.027), whereas the right DTI-ALPS index correlated with MoCA (*r* = 0.232, *p*_FDR_ = 0.027), memory (*r* = 0.295, *p*_FDR_ = 0.011), executive function (*r* = 0.232, *p*_FDR_ = 0.027) and visuospatial abilities (*r* = 0.223, *p*_FDR_ = 0.046). Neither serum vitamin B12 nor Hcy showed significant associations with any cognitive measure (*p*_FDR_ > 0.05). Detailed results are presented in Supplementary Table S4.

### 3.7 The relationship between serum folate levels, DTI-ALPS index, and cognitive function in AD group

**Figure 6:**
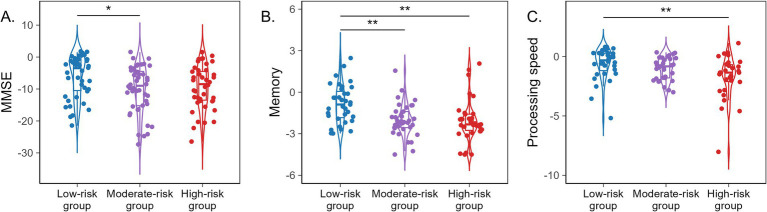
Box plots illustrate cognitive differences across the low-risk group, moderate-risk group, and high-risk group. **(A)** MMSE. **(B)** Memory. **(C)** Processing speed. *p < 0.05; **p < 0.01; ***p < 0.001.

To further explore the joint effects of folate status and glymphatic function on cognition, we performed multiple linear regression analysis by including folate, the DTI-ALPS index, and their interaction term (folate × ALPS) in the model, with adjustment for age, sex, education years, and APOE ε4 status, to examine whether folate and glymphatic function interact to influence various cognitive domains. The results revealed significant folate × ALPS interactions for the memory domain (β = 0.17, *p* = 0.031) and for MoCA (β = 0.184, *p* = 0.029), suggesting that the effect of folate on cognitive function may depend on the level of glymphatic function. The multiple linear regression equation is presented below, and the full regression results are provided in Supplementary Table S5. AD patients were stratified according to the median serum folate concentration and median DTI-ALPS index into three subgroups: high-risk group (low folate levels and low DTI-ALPS index), moderate-risk group (low folate levels and high DTI-ALPS index or high folate levels and low DTI-ALPS index), and low-risk group (high folate levels and high DTI-ALPS index). Supplementary Table S6 presents demographic characteristics and clinical data for all subjects across subgroups. *Post hoc* comparisons revealed that compared with the low-risk group, the medium-risk group showed significantly poorer performance in MMSE (*p* = 0.021) and memory (*p* = 0.003) domains, while the high-risk group demonstrated significantly worse performance in memory (*p* = 0.001) and information processing speed (*p* = 0.005). No statistically significant differences were found in MoCA in *post hoc* tests (Figure 6).


$$\begin{array}{l} \mathrm{Cognition}=\beta 0+\beta 1*\mathrm{Folate}+\beta 2*\mathrm{DTI}-\mathrm{ALPS}\\ +\beta 3*\left(\mathrm{Folate}*\mathrm{DTI}-\mathrm{ALPS}\right)+\beta 4*\mathrm{Age}+\beta 5*\mathrm{Sex}\\ +\beta 6*\mathrm{Education}+\beta 7*\mathrm{APOE status}+\varepsilon \end{array}$$

## 4 Discussion

This study is the first to systematically characterize the interrelationships among circulating biomarkers of OCM (folate, vitamin B12, and Hcy), glymphatic function as measured by DTI-ALPS index, and cognitive performance. We found that both the DTI-ALPS index and serum concentrations of folate and vitamin B12 were significantly lower in the AD-D group than in cognitively NC, with similar patterns observed in female and late-life subgroups. Second, within the AD cohort (AD-MCI and AD-D combined), serum folate levels correlated positively, whereas Hcy levels correlated negatively, with the DTI-ALPS index. Third, both serum folate and the DTI-ALPS index were significantly associated with performance on global and domain-specific cognitive measures (e.g., MoCA and executive function). Moreover, compared with the low-risk group (high folate and high DTI-ALPS index), participants in the moderate-risk group (low folate with high DTI-ALPS index or high folate with low DTI-ALPS index) showed poorer MMSE and memory performance, while the high-risk group (low folate and low DTI-ALPS index) exhibited marked deficits in memory and processing speed.

A substantial body of cross-sectional and longitudinal evidence has consistently demonstrated that OCM dysfunction markedly increases the risk of AD-related cognitive impairment (9). Reduced serum folate has been identified in multiple studies as an independent risk factor for accelerated cognitive decline (13), and both CSF and serum folate levels have been found to be significantly lower in AD patients than cognitively normal controls (14). These observations reinforce the notion that folate deficiency may contribute causally to AD onset and progression through mechanisms involving impaired methylation, neuroinflammation, and oxidative stress. Consistent with these reports, our study found that serum folate and vitamin B12 levels were significantly lower in AD patients than in the NC group, even though both vitamins remained within the conventional normal range. In contrast, Hcy concentrations did not differ significantly across the three groups, although levels remained universally elevated (plasma Hcy > 10–15 μmol/L defines hyperhomocysteinemia) (32). Interestingly, this age-related metabolic pattern likely reflects the high prevalence of hyperhomocysteinemia in the elderly population. Previous studies have shown that Hcy concentrations increase markedly after the age of 50 (33). Similar patterns were observed in our stratified analyses: in both the female and late-life subgroups, AD patients exhibited significantly lower serum folate, vitamin B12, and the whole-brain and bilateral ALPS indices compared with the NC group, whereas no significant difference was observed in Hcy. Epidemiological evidence consistently indicates that women and older adults are at intrinsically higher risk of developing AD (34–36). Approximately two-thirds of AD patients are women, and females generally face a greater lifetime threat of age-related disorders (34). The lifetime risk of AD dementia is roughly 41.9% for women versus 33.6% for men (35). The prevalence of AD dementia among people aged 60 years and older increases with age (36). The consistency of the stratified analysis results supports the robustness of our primary findings and also suggests that women and elderly individuals may be priority populations for future research and nutritional interventions. However, it should be emphasized that these results should be regarded as exploratory findings and may serve as a reference for subsequent studies. In this study, we further observed that, within the AD group, serum folate was significantly and positively associated with both global (MMSE, MoCA) and domain-specific (executive function) cognitive measures, suggesting that adequate folate status may help preserve cognitive integrity. By contrast, neither Hcy nor vitamin B12 showed statistically significant associations with cognitive performance, although their correlations trended negative and positive, respectively.

On the other hand, growing neuroimaging evidence supports impaired glymphatic function as another key mechanism in AD (5, 37). A large body of cross-sectional studies has reported that the DTI-ALPS index is markedly lower in patients with AD or MCI than in healthy elderly controls, and the magnitude of this reduction closely parallels the severity of cognitive impairment (38). The current results support these findings: significantly lower whole-brain and bilateral ALPS indices were observed in AD-D patients versus the NC group, potentially indicative of altered cerebral waste clearance dynamics in AD. Moreover, the DTI-ALPS index was positively correlated with MoCA scores and several critical cognitive domains, including memory, executive function, attention, and visuospatial abilities. These findings support the hypothesis that glymphatic dysfunction may exacerbate cognitive decline in AD. We also observed a left–right difference in the DTI-ALPS index across the AD cohort. This finding is consistent with a recent study proposing that glymphatic dysfunction originates in the left hemisphere and spreads to the right as AD progresses (39). Earlier research has also reported more severe metabolic dysfunction in the left hemisphere than in the right hemisphere of individuals with AD (40).

A recent study investigating the association between dietary nutrients and glymphatic activity in healthy participants found no correlation between the DTI-ALPS index and serum folate (41). In contrast, our study revealed a significant positive correlation between the DTI-ALPS index and serum folate, which may be attributed to differences in the study populations. Our cohort was relatively older and exhibited significantly worse overall cognition, and both age and cognitive status have been previously shown to be associated with the DTI-ALPS index (42). In addition, we observed a significant negative correlation between serum Hcy and the DTI-ALPS index, consistent with a recent cross-sectional case–control study (43). Interestingly, this association was confined to the AD group and absent in the NC group (Supplementary Table S7). This parallels recent findings that no relationship between folate levels and the DTI-ALPS index was detected in healthy individuals (41), whereas a significant negative correlation between Hcy and the DTI-ALPS index was documented in H-type hypertensive patients, who are characterized by elevated Hcy and in whom this phenotype constitutes an independent risk factor for vascular cognitive impairment (43). Of note, Hcy concentrations in our cohort were also relatively high. No significant correlation was observed between vitamin B12 and the DTI-ALPS index, consistent with previous studies establishing no clear link between them. It should be noted that although the serum folate and vitamin B12 levels observed in our AD patients were significantly lower than those in the control group, most individuals still fell within the conventional normal range. This suggests that even subtle fluctuations within the normal range, when combined with other factors such as glymphatic dysfunction, may be associated with cognitive decline, rather than indicating overt nutritional deficiency. Collectively, these results suggest that folate and Hcy may play a more direct or pivotal role in modulating glymphatic function, whereas vitamin B12 deficiency might influence brain health through slower or distinct mechanistic pathways. It is worth noting that the correlation coefficients observed in our study among OCM biomarkers, the DTI-ALPS index, and cognitive performance (r ranging from −0.4 to 0.4), while explaining only a modest proportion of the variance, fall within the range commonly reported in the literature. For instance, Lv et al. reported correlations of *r* = 0.345 between serum folate and MMSE scores, and *r* = 0.355 between serum folate and MoCA scores (75). Ota et al. found that the associations between the DTI-ALPS index and nutritional indicators such as B-vitamins were generally weak, with correlation coefficients ranging from −0.1 to 0.2 (76). Similarly, Zhang and colleagues observed a correlation of *r* = 0.173 between the DTI-ALPS index and MoCA scores (77). Taken together, our results are compatible with the possibility of a relationship between impaired glymphatic clearance and reduced serum folate levels in AD.

Nevertheless, the biological basis underlying the relationship between circulating biomarkers of OCM and glymphatic drainage in AD remains unclear. Previous studies have demonstrated that folate serves as a methyl donor; insufficient folate availability disrupts DNA methylation, a key cellular regulatory mechanism in which a methyl group (CH3) is covalently added to cytosine residues adjacent to guanine (CpG sites) by DNA methyltransferases (44). Genome-wide analyses have also revealed abundant non-CpG methylation (CpA, CpT and CpC) throughout the genome, with neuronal cells exhibiting particularly high levels (45). Such atypical methylation has also been detected in AD-related genes, implying that folate may play a pivotal role in AD and other neurodegenerative disorders (46). Folate deficiency can also lead to increases or decreases in gene expression, accompanied by alterations in DNA methylation and subsequent changes in protein synthesis (47). Beyond epigenetic regulation, folate exerts its antioxidant properties and neuroprotective potential (48). As a cofactor for glutathione (GSH)-related enzymes, folate enhances antioxidant defenses and mitigates oxidative stress (48). Supplementation with folate has been shown to modulate transcriptional regulation of nicotinamide adenine dinucleotide phosphate (NADPH) oxidase, which may partly account for its direct antioxidant effects (49). Additionally, folate also indirectly reduces oxidative stress by lowering Hcy levels (50). In contrast, Hcy acts as a pro-oxidant by activating protease-activated receptors (PARs) and inhibiting endothelial nitric oxide synthase (eNOS) (51), leading to increased ROS generation and subsequent cellular damage and death (52). Furthermore, folate is essential for neurotransmitter synthesis (11), myelination (12), and lifelong neuronal growth and repair (13). The glymphatic system removes ROS and aberrant waste proteins; conversely, increased oxidative stress and metabolic waste impair glymphatic function (41). By counteracting these processes, folate may help sustain glymphatic activity. In our stratified study, patients in the high-risk group (low folate and low DTI-ALPS index) and moderate-risk groups (either low folate with high DTI-ALPS index or high folate with low DTI-ALPS index), exhibited significantly poorer cognitive performance than those in the low-risk group (high folate and high DTI-ALPS index). Specifically, the moderate-risk group performed worse on the MMSE (*p* = 0.021) and memory domain (*p* = 0.003), whereas the high-risk group showed pronounced impairments in memory (*p* = 0.001) and information processing speed (*p* = 0.005). These findings may suggest a synergistic effect of folate and the DTI-ALPS index on cognitive performance. In summary, folate deficiency and glymphatic clearance failure may jointly drive the pathogenesis of AD, synergistically amplifying AD pathology from both metabolic and eliminative perspectives and ultimately precipitating cognitive decline. These findings may lay a theoretical foundation for future exploration of comprehensive AD treatment strategies combining nutritional modulation and glymphatic enhancement.

This study has several limitations. First, the majority of participants were diagnosed using the 2011 NIA-AA clinical criteria, with only a minority classified according to the 2018 ATN research framework. The lack of biomarker confirmation (CSF Aβ/tau or amyloid PET) may introduce potential misclassification, particularly within the MCI cohort, where non-AD pathologies may account for cognitive impairment. Future studies incorporating biomarker-defined cohorts are essential to validate our findings. Second, we employed the ALPS index as a surrogate marker of glymphatic function. Although widely used in neuroimaging, this metric has notable constraints; recent critiques suggest that it primarily reflects radial water diffusivity adjacent to the lateral ventricular body, rather than encompassing the broader dynamics of glymphatic clearance, and thus may oversimplify its relationship with overall drainage capacity (53, 54). Future studies incorporating additional CSF dynamics measures, such as CSF-BOLD coupling, or applying lymphatic-specific MRI techniques to directly capture glymphatic function, may help to further substantiate our conclusions. Third, our assessment of OCM relied exclusively on single-time-point measurements of serum folate, vitamin B12, and Hcy. While these circulating biomarkers are widely used in clinical research, they primarily reflect recent dietary intake and metabolic fluctuations rather than long-term nutritional status. The absence of complementary measures, such as erythrocyte folate concentrations (which better reflect tissue stores), detailed dietary records, or genetic polymorphisms affecting folate metabolism (e.g., MTHFR C677T) limits our ability to draw definitive conclusions about chronic metabolic dysfunction. Future studies incorporating comprehensive nutritional assessments and multiple complementary biomarkers are warranted to validate our findings. Fourth, although we adjusted for major demographic and clinical covariates (e.g., age, sex, education years, and APOE genotype), we cannot completely rule out the potential influence of unmeasured confounding factors. These factors include, but are not limited to, renal function, comprehensive nutritional status, sleep quality, and subtle motion artifacts during image acquisition. For example, renal dysfunction may indirectly affect folate metabolism by elevating Hcy levels, while poor sleep quality has been shown to impair glymphatic clearance function. In the present study, we were unable to obtain data to control for these variables. Therefore, future studies should incorporate comprehensive assessments of these factors to more accurately delineate the role of folate and glymphatic function in cognitive decline. Finally, this study employed a cross-sectional design, which precludes determination of causal relationships between folate levels, the DTI-ALPS index, and cognitive function. While folate deficiency and glymphatic system dysfunction may contribute to cognitive decline, they could also be influenced by other confounding factors. For instance, in our study, the lower folate levels observed in AD patients may reflect inadequate dietary intake secondary to cognitive impairment, rather than serving as a primary etiological factor. Future longitudinal, large-scale, multimodal studies are warranted to establish causal pathways and facilitate clinical translation.

## 5 Conclusion

Our findings showed that the DTI-ALPS index decreased with lower serum folate levels, with a linear association observed between the two. We further observed that the combined presence of lower folate levels and reduced DTI-ALPS index was associated with poorer cognitive performance. These results suggest a potential link between folate status and glymphatic function in relation to cognition. Whether these associations have clinical implications requires confirmation in longitudinal and mechanistic studies.

## Data Availability

The original contributions presented in the study are included in the article/Supplementary material, further inquiries can be directed to the corresponding authors.

## Footnotes

1
https://www.r-project.org/
