# Supplementary material for: Association between circulating biomarkers of one-carbon metabolism and glymphatic system function in cognitive decline of Alzheimer’s disease
Source: Front Neurol. 2026 May 11;17:1779257. doi: 10.3389/fneur.2026.1779257 (PMC13199100; doi:10.3389/fneur.2026.1779257)
Supplement: Supplementary file 1 [file Table_1.docx]

| **Cognitive function** | **Tests included** | **Cronbach's α** |
| --- | --- | --- |
| **Memory** | **AVLT (immediate recall, short-term delayed recall, long-term delayed recall, recognition)** | **0.915** |
| **Executive function** | **SCWT-C, TMT-B, VFT** | **0.731** |
| **Attention** | **DST (forward, backward)** | **0.766** |
| **Processing speed** | **TMT-A, SCWT-A, SCWT-B** | **0.728** |

**Table S1.** Internal consistency
