# Supplementary material for: Association between circulating biomarkers of one-carbon metabolism and glymphatic system function in cognitive decline of Alzheimer’s disease
Source: Front Neurol. 2026 May 11;17:1779257. doi: 10.3389/fneur.2026.1779257 (PMC13199100; doi:10.3389/fneur.2026.1779257)
Supplement: Supplementary file 2 [file Table_2.docx]

**Table S2.** Hierarchical analysis.

| **Stratification** | **variables** | **NC group** | **AD group** | **p value** |
| --- | --- | --- | --- | --- |
| **Total (n=210)** | DTI-ALPS index | 1.25 ± 0.13 | 1.18 ± 0.14 | **< 0.001^a^** |
|  | Left DTI-ALPS index | 1.25 ± 0.12 | 1.18 ± 0.14 | **< 0.001^a^** |
|  | Right DTI-ALPS index | 1.25 ± 0.15 | 1.19 ± 0.16 | **0.003^a^** |
|  | Folate, ng/mL | 12.22 (7.73, 14.45) | 9.28 (6.96, 12.52) | **0.020^b^** |
|  | Vitamin B12, pg/mL | 599.97 ± 206.65 | 531.96 ± 181.06 | **0.003^a^** |
|  | Homocysteine, μmol/L | 16.69 (14.20, 18.94) | 17.04 (14.43, -21.08) | 0.267^b^ |
| **Female (n=125)** | DTI-ALPS index | 1.30 ± 0.10 | 1.24 ± 0.13 | **0.001^a^** |
|  | Left DTI-ALPS index | 1.29 ± 0.10 | 1.23 ± 0.13 | **< 0.001^a^** |
|  | Right DTI-ALPS index | 1.31 ± 0.11 | 1.25 ± 0.14 | **0.004^a^** |
|  | Folate, ng/mL | 12.59 ± 3.82 | 10.29 ± 3.87 | **0.005^a^** |
|  | Vitamin B12, pg/mL | 653.79 ± 198.40 | 573.10 ± 185.17 | **0.003^a^** |
|  | Homocysteine, μmol/L | 15.48 (12.84, 18.43) | 16.09 (13.99, 20.32) | 0.125^b^ |
| **Male (n=85)** | DTI-ALPS index | 1.17 ± 0.14 | 1.11 ± 0.13 | 0.132^a^ |
|  | Left DTI-ALPS index | 1.19 ± 0.13 | 1.12 ± 0.14 | 0.101^a^ |
|  | Right DTI-ALPS index | 1.15 (1.03, 1.25) | 1.12 (1.01, 1.22) | 0.512^b^ |
|  | Folate, ng/mL | 8.32 (5.91, 13.87) | 9.04 (6.70, 12.11) | 0.714^b^ |
|  | Vitamin B12, pg/mL | 512.83 ± 193.52 | 479.07 ± 162.39 | 0.377^a^ |
|  | Homocysteine, μmol/L | 18.11 (15.90, 22.21) | 17.58 (15.26, 22.68) | 0.582^b^ |
| **Late-life (n=123)** | DTI-ALPS index | 1.21 ± 0.13 | 1.16 ± 0.13 | **0.021^a^** |
|  | Left DTI-ALPS index | 1.20 ± 0.11 | 1.15 ± 0.13 | **0.018^a^** |
|  | Right DTI-ALPS index | 1.22 ± 0.16 | 1.16 ± 0.14 | **0.039^a^** |
|  | Folate, ng/mL | 12.77 (6.56, 16.34) | 9.03 (6.55, 11.85) | **0.046^b^** |
|  | Vitamin B12, pg/mL | 605.51 ± 203.10 | 533.00 ± 177.89 | **0.026^a^** |
|  | Homocysteine, μmol/L | 16.47 (13.69, 19.47) | 17.38 (14.59, 22.64) | 0.450^b^ |
| **Mild-life (n=87)** | DTI-ALPS index | 1.29 ± 0.11 | 1.23 ± 0.16 | 0.055^a^ |
|  | Left DTI-ALPS index | 1.30 ± 0.11 | 1.23 ± 0.15 | **0.048^a^** |
|  | Right DTI-ALPS index | 1.29 ± 0.13 | 1.24 ± 0.17 | 0.094^a^ |
|  | Folate, ng/mL | 11.84 (9.07, 14.20) | 10.34 (8.00, 13.84) | 0.425^b^ |
|  | Vitamin B12, pg/mL | 10.34 ± 213.98 | 530.17 ± 188.32 | **0.024^a^** |
|  | Homocysteine, μmol/L | 16.69 (14.20, 18.94) | 16.16 (14.23, 19.70) | 0.556^b^ |

Notes: NC group, normal control group; AD group, combined AD-D and AD-MCI patients; Late-life, ≥60 years; Mid-life, <60 years; DTI-ALPS, diffusion tensor image analysis along the perivascular space.

^a^ p-value for comparison between the NC and low-cognition groups using independent-samples t-test.

^b^ p-value for comparison between the NC and low-cognition groups using Mann–Whitney U test.
