# Supplementary material for: Association between circulating biomarkers of one-carbon metabolism and glymphatic system function in cognitive decline of Alzheimer’s disease
Source: Front Neurol. 2026 May 11;17:1779257. doi: 10.3389/fneur.2026.1779257 (PMC13199100; doi:10.3389/fneur.2026.1779257)
Supplement: Supplementary file 3 [file Table_3.docx]

**Table S3.** Detailed data for the scatter plot.

| **Variables** | **Variables** | **Correlation coefficient** | **p value** | **p_FDR_ value** |
| --- | --- | --- | --- | --- |
| Folate | DTI-ALPS index | 0.212 | **0.016^a^** | **0.032^a^** |
|  | Left DTI-ALPS index | 0.209 | **0.018^a^** | **0.032^a^** |
|  | Right DTI-ALPS index | 0.196 | **0.027^a^** | **0.041^a^** |
| Vitamin B12 | DTI-ALPS index | 0.138 | 0.111^a^ | 0.125^a^ |
|  | Left DTI-ALPS index | 0.095 | 0.275^a^ | 0.275^a^ |
|  | Right DTI-ALPS index | 0.166 | 0.054^a^ | 0.069^a^ |
| Homocysteine | DTI-ALPS index | -0.299 | **<0.001^b^** | **<0.001^b^** |
|  | Left DTI-ALPS index | -0.262 | **0.002^b^** | **0.006^b^** |
|  | Right DTI-ALPS index | -0.287 | **<0.001^b^** | **<0.001^b^** |

Note:DTI-ALPS, diffusion tensor image analysis along the perivascular space.

^a^ p-value obtained from Pearson correlation analysis. ^b^ p-value obtained from Spearman correlation analysis.
