# Supplementary material for: Association between circulating biomarkers of one-carbon metabolism and glymphatic system function in cognitive decline of Alzheimer’s disease
Source: Front Neurol. 2026 May 11;17:1779257. doi: 10.3389/fneur.2026.1779257 (PMC13199100; doi:10.3389/fneur.2026.1779257)
Supplement: Supplementary file 4 [file Table_4.docx]

**Table S4.** Detailed data for the heatmap.

| **Variables** | **Variables** | **Correlation coefficient** | **p value** | **p_FDR_ value** |
| --- | --- | --- | --- | --- |
| Folate | MMSE | 0.258 | **0.004** | **0.019** |
|  | MoCA | 0.238 | **0.012** | **0.030** |
|  | Memory | 0.222 | **0.027** | 0.057 |
|  | Executive functions | 0.238 | **0.012** | **0.030** |
|  | Attention | 0.165 | 0.081 | 0.142 |
|  | Processing speed | 0.171 | 0.312 | 0.397 |
|  | Visuospatial abilities | 0.144 | 0.158 | 0.255 |
| Vitamin B12 | MMSE | 0.043 | 0.636 | 0.676 |
|  | MoCA | 0.161 | 0.081 | 0.142 |
|  | Memory | 0.133 | 0.176 | 0.264 |
|  | Executive functions | 0.161 | 0.081 | 0.142 |
|  | Attention | 0.109 | 0.238 | 0.312 |
|  | Processing speed | 0.072 | 0.663 | 0.679 |
|  | Visuospatial abilities | 0.063 | 0.526 | 0.613 |
| Homocysteine | MMSE | 0.052 | 0.555 | 0.613 |
|  | MoCA | -0.054 | 0.553 | 0.613 |
|  | Memory | -0.121 | 0.202 | 0.274 |
|  | Executive functions | -0.054 | 0.553 | 0.613 |
|  | Attention | -0.042 | 0.644 | 0.676 |
|  | Processing speed | -0.125 | 0.436 | 0.539 |
|  | Visuospatial abilities | 0.036 | 0.707 | 0.707 |
| DTI-ALPS index | MMSE | 0.197 | **0.023** | 0.051 |
|  | MoCA | 0.282 | **0.001** | **0.007** |
|  | Memory | 0.339 | **<0.001** | **<0.001** |
|  | Executive functions | 0.283 | **0.001** | **0.007** |
|  | Attention | 0.245 | **0.006** | **0.025** |
|  | Processing speed | 0.219 | 0.169 | 0.263 |
|  | Visuospatial abilities | 0.255 | **0.007** | **0.027** |
| Left DTI-ALPS index | MMSE | 0.221 | **0.011** | **0.030** |
|  | MoCA | 0.299 | **<0.001** | **<0.001** |
|  | Memory | 0.343 | **<0.001** | **<0.001** |
|  | Executive functions | 0.299 | **<0.001** | **<0.001** |
|  | Attention | 0.279 | **0.002** | **0.011** |
|  | Processing speed | 0.211 | 0.186 | 0.270 |
|  | Visuospatial abilities | 0.255 | **0.008** | **0.027** |
| Right DTI-ALPS index | MMSE | 0.149 | 0.087 | 0.146 |
|  | MoCA | 0.232 | **0.009** | **0.027** |
|  | Memory | 0.295 | **0.002** | **0.011** |
|  | Executive functions | 0.232 | **0.009** | **0.027** |
|  | Attention | 0.182 | **0.043** | 0.086 |
|  | Processing speed | 0.208 | 0.193 | 0.270 |
|  | Visuospatial abilities | 0.223 | **0.020** | **0.046** |

Note: MMSE, Mini-Mental State Examination; MoCA, Montreal Cognitive Assessment. DTI-ALPS, diffusion tensor image analysis along the perivascular space.
