# Supplementary material for: Association between circulating biomarkers of one-carbon metabolism and glymphatic system function in cognitive decline of Alzheimer’s disease
Source: Front Neurol. 2026 May 11;17:1779257. doi: 10.3389/fneur.2026.1779257 (PMC13199100; doi:10.3389/fneur.2026.1779257)
Supplement: Supplementary file 5 [file Table_5.docx]

**Table S5.** Multiple linear regression

| **Dependent variable** | **Variables** | **Variables** | **Unstandardized coefficient** | | **Standardized coefficient** | **t-value** | **p-value** | **95% Confidence Interval** | **R2** | **Adjusted R²** | **F-value** |
| --- | --- | --- | --- | --- | --- | --- | --- | --- | --- | --- | --- |
|  |  |  | **β** | **SE** |  |  |  |  |  |  |  |
| **MMSE** | **Independent variable** | **Folate** | 0.412 | 0.159 | 0.218 | 2.597 | 0.011 | (0.098, 0.726) | 0.257 | 0.212 | 5.719 |
|  |  | **DTI-ALPS** | 8.05 | 4.885 | 0.163 | 1.648 | 0.102 | (-1.625, 17.726) |  |  |  |
|  |  | **Folate*DTI-ALPS** | 2.049 | 1.135 | 0.147 | 1.805 | 0.074 | (-0.199, 4.297) |  |  |  |
|  | **Covariate** | **Age** | 0.057 | 0.084 | 0.063 | 0.677 | 0.499 | (-0.11, 0.224) |  |  |  |
|  |  | **Sex** | 1.205 | 1.312 | 0.084 | 0.919 | 0.36 | (-1.393, 3.803) |  |  |  |
|  |  | **Education** | 0.62 | 0.141 | 0.373 | 4.413 | **<0.001** | (0.342, 0.899) |  |  |  |
|  |  | **APOE** | -3.29 | 1.249 | -0.223 | -2.634 | 0.01 | (-5.763, -0.816) |  |  |  |
| **MoCA** | **Independent variable** | **Folate** | 0.124 | 0.053 | 0.2 | 2.333 | **0.021** | (0.019, 0.23) | 0.29 | 0.244 | 6.29 |
|  |  | **DTI-ALPS** | 4.516 | 1.652 | 0.268 | 2.734 | **0.007** | (1.242, 7.791) |  |  |  |
|  |  | **Folate*DTI-ALPS** | 0.868 | 0.391 | 0.184 | 2.218 | **0.029** | (0.092, 1.643) |  |  |  |
|  | **Covariate** | **Age** | 0.004 | 0.028 | 0.012 | 0.131 | 0.896 | (-0.052, 0.059) |  |  |  |
|  |  | **Sex** | 0.544 | 0.435 | 0.115 | 1.251 | 0.214 | (-0.318, 1.407) |  |  |  |
|  |  | **Education** | 0.211 | 0.049 | 0.362 | 4.293 | **<0.001** | (0.114, 0.308) |  |  |  |
|  |  | **APOE** | -0.969 | 0.425 | -0.196 | -2.281 | **0.025** | (-1.811, -0.127) |  |  |  |
| **Memory** | **Independent variable** | **Folate** | 0.059 | 0.03 | 0.157 | 1.977 | 0.051 | (0, 0.118) | 0.47 | 0.431 | 12.03 |
|  |  | **DTI-ALPS** | 3.107 | 0.902 | 0.309 | 3.447 | **0.001** | (1.317, 4.897) |  |  |  |
|  |  | **Folate*DTI-ALPS** | 0.465 | 0.213 | 0.17 | 2.185 | **0.031** | (0.042, 0.887) |  |  |  |
|  | **Covariate** | **Age** | -0.031 | 0.015 | -0.173 | -2.017 | **0.047** | (-0.062, 0) |  |  |  |
|  |  | **Sex** | -0.014 | 0.24 | -0.005 | -0.059 | 0.953 | (-0.49, 0.462) |  |  |  |
|  |  | **Education** | 0.081 | 0.027 | 0.229 | 2.972 | **0.004** | (0.027, 0.135) |  |  |  |
|  |  | **APOE** | -1.093 | 0.237 | -0.366 | -4.622 | **<0.001** | (-1.563, -0.624) |  |  |  |
| **Executive function** | **Independent variable** | **Folate** | 0.038 | 0.058 | 0.127 | 0.659 | 0.514 | (-0.079, 0.155) | 0.258 | 0.1 | 1.638 |
|  |  | **DTI-ALPS** | 1.665 | 1.714 | 0.206 | 0.972 | 0.338 | (-1.821, 5.152) |  |  |  |
|  |  | **Folate*DTI-ALPS** | 0.001 | 0.373 | 0.001 | 0.004 | 0.997 | (-0.757, 0.76) |  |  |  |
|  | **Covariate** | **Age** | -0.037 | 0.024 | -0.282 | -1.52 | 0.138 | (-0.087, 0.013) |  |  |  |
|  |  | **Sex** | 0.368 | 0.408 | 0.16 | 0.904 | 0.373 | (-0.461, 1.198) |  |  |  |
|  |  | **Education** | 0.022 | 0.051 | 0.074 | 0.427 | 0.672 | (-0.083, 0.127) |  |  |  |
|  |  | **APOE** | -0.486 | 0.489 | -0.188 | -0.994 | 0.327 | (-1.481, 0.509) |  |  |  |
| **Attention** | **Independent variable** | **Folate** | 0.037 | 0.025 | 0.122 | 1.49 | 0.139 | (-0.012, 0.086) | 0.354 | 0.312 | 8.447 |
|  |  | **DTI-ALPS** | 1.987 | 0.776 | 0.238 | 2.562 | **0.012** | (0.45, 3.524) |  |  |  |
|  |  | **Folate*DTI-ALPS** | 0.349 | 0.183 | 0.151 | 1.905 | 0.059 | (-0.014, 0.713) |  |  |  |
|  | **Covariate** | **Age** | 0.003 | 0.013 | 0.022 | 0.243 | 0.808 | (-0.023, 0.029) |  |  |  |
|  |  | **Sex** | 0.37 | 0.204 | 0.158 | 1.818 | 0.072 | (-0.033, 0.774) |  |  |  |
|  |  | **Education** | 0.149 | 0.023 | 0.524 | 6.521 | **<0.001** | (0.104, 0.195) |  |  |  |
|  |  | **APOE** | -0.277 | 0.199 | -0.114 | -1.394 | 0.166 | (-0.671, 0.117) |  |  |  |
| **Processing speed** | **Independent variable** | **Folate** | 0.088 | 0.033 | 0.275 | 2.716 | **0.008** | (0.024, 0.153) | 0.247 | 0.183 | 3.885 |
|  |  | **DTI-ALPS** | 1.648 | 1.05 | 0.185 | 1.57 | 0.12 | (-0.44, 3.737) |  |  |  |
|  |  | **Folate*DTI-ALPS** | 0.453 | 0.248 | 0.187 | 1.828 | 0.071 | (-0.04, 0.947) |  |  |  |
|  | **Covariate** | **Age** | -0.021 | 0.018 | -0.139 | -1.191 | 0.237 | (-0.056, 0.014) |  |  |  |
|  |  | **Sex** | 0.379 | 0.251 | 0.158 | 1.512 | 0.134 | (-0.12, 0.878) |  |  |  |
|  |  | **Education** | 0.047 | 0.03 | 0.16 | 1.578 | 0.118 | (-0.012, 0.107) |  |  |  |
|  |  | **APOE** | -0.02 | 0.257 | -0.008 | -0.079 | 0.937 | (-0.531, 0.491) |  |  |  |
| **Visuospatial abilities** | **Independent variable** | **Folate** | 0.058 | 0.048 | 0.123 | 1.217 | 0.226 | (-0.037, 0.153) | 0.14 | 0.076 | 2.18 |
|  |  | **DTI-ALPS** | 2.678 | 1.398 | 0.22 | 1.915 | 0.058 | (-0.098, 5.453) |  |  |  |
|  |  | **Folate*DTI-ALPS** | 0.317 | 0.331 | 0.094 | 0.957 | 0.341 | (-0.34, 0.974) |  |  |  |
|  | **Covariate** | **Age** | 0.048 | 0.024 | 0.219 | 1.996 | **0.049** | (0, 0.096) |  |  |  |
|  |  | **Sex** | -0.051 | 0.368 | -0.015 | -0.139 | 0.89 | (-0.782, 0.68) |  |  |  |
|  |  | **Education** | 0.127 | 0.043 | 0.289 | 2.941 | **0.004** | (0.041, 0.213) |  |  |  |
|  |  | **APOE** | -0.063 | 0.368 | -0.017 | -0.171 | 0.865 | (-0.794, 0.668) |  |  |  |

Notes: MMSE, Mini-Mental State Examination; MoCA, Montreal Cognitive Assessment. DTI-ALPS, diffusion tensor image analysis along the perivascular space
