# Supplementary material for: Association between circulating biomarkers of one-carbon metabolism and glymphatic system function in cognitive decline of Alzheimer’s disease
Source: Front Neurol. 2026 May 11;17:1779257. doi: 10.3389/fneur.2026.1779257 (PMC13199100; doi:10.3389/fneur.2026.1779257)
Supplement: Supplementary file 6 [file Table_6.docx]

**Table S6.** Relationship between serum folate levels, DTI-ALPS Index, and cognitive function.

| **Characteristics** | **Low-risk group(n=41)** | **Moderate-risk group(n=46)** | **High-risk group(n=41)** | **p value** |
| --- | --- | --- | --- | --- |
| Age, (years) | 60 (54, 67.5) | 62.5 (58, 71) | 67 (59, 73.5) | **0.016** |
| Sex, n (%) |  |  |  | **0.003** |
| Female | 30 (73.2%) | 27 (58.7%) | 15 (36.6%) |  |
| Male | 11 (26.8%) | 19 (41.3%) | 26 (63.4%) |  |
| Education, (years) | 8 (4, 9.5) | 8 (4, 11) | 9 (7, 12) | 0.319 |
| BMI, kg/m^2^ | 22.89 (20.82, 25.05) | 22.94(20.24, 24.58) | 23.31(20.67, 24.11) | 0.961 |
| **Vascular risk factors** |  |  |  |  |
| Hypertension, n (%) | 11 (28.2%) | 13 (28.3%) | 20 (52.6%) | **0.033** |
| Diabetes, n (%) | 4 (9.8%) | 7 (15.2%) | 5 (12.2%) | 0.742 |
| Hyperlipidemia, n (%) | 2 (4.9%) | 10 (21.7%) | 7 (17.1%) | 0.078 |
| Smoking, n (%) | 5 (12.2%) | 5 (10.9%) | 19 (46.3%) | **< 0.001** |
| Drinking, n (%) | 5 (12.2%) | 15 (32.6%) | 18 (43.9%) | **0.006** |
| **APOE gene** |  |  |  | 0.801 |
| ε4 carriers, n (%) | 14 (35%) | 18 (40%) | 13 (33.3%) |  |
| ε4 non-carriers, n (%) | 26 (65%) | 27 (60%) | 26 (66.7%) |  |
| **Cognitive function** |  |  |  |  |
| MMSE | -3.499 (-11.49, -0.49) | -8.99 (-15.49, -4.49) | -8.489 (-13.49, -4.49) | **0.014** |
| MoCA | -1.84 (-4.56, -0.13) | -3.20 (-5.25, -1.49) | -3.88 (-4.90, -1.834) | **0.033** |
| Memory | -0.89 (-1.91, 0.23) | -2.16 (-2.58, -1.40) | -2.33 (-2.82, -1.58) | **< 0.001** |
| Executive functions | -0.38 (-0.89, 0.46) | -0.23 (-0.72, 0.18) | -0.47 (-1.30, 0.22) | 0.757 |
| Attention | -0.37 ± 1.05 | -0.86 ± 1.12 | -0.78 ± 1.32 | 0.111 |
| Processing speed | -0.32 (-1.35, 0.29) | -0.82 (-1.82, -0.11) | -1.35 (-2.31, -0.61) | **0.006** |
| Visuospatial abilities | -0.86 (-1.52, 0.75) | -1.12 (-3.00, 0.22) | -1.66(-3.00, -0.32) | 0.204 |
| **DTI-ALPS** |  |  |  |  |
| Left DTI-ALPS index | 1.29 (1.22, 1.34) | 1.19(1.11, 1.31) | 1.08 (0.98, 1.12) | **< 0.001** |
| Right DTI-ALPS index | 1.30 ± 0.97 | 1.20 ± 0.15 | 1.06 ± 0.11 | **< 0.001** |
| DTI-ALPS index | 1.29 (1.23, 1.34) | 1.20(1.12, 1.28) | 1.08 (0.98, 1.15) | **< 0.001** |
| **Serological markers** |  |  |  |  |
| Folate, ng/mL | 12.24 (10.57, 15.44) | 9.55 (7.01, 12.89) | 6.92 (5.74, 8.26) | **< 0.001** |
| Vitamin B12, pg/mL | 582.39 ± 182.53 | 511.67 ± 187.25 | 504.29 ± 165.69 | 0.099 |
| Homocysteine, μmol/L | 15.44 (14.01, 18.54) | 16.96 (13.68, 20.43) | 20.61 (16.09, 26.37) | **< 0.001** |

Notes: Continuous variables were assessed for intergroup differences using one-way ANOVA or Kruskal-Wallis tests. Categorical variables were compared using χ² tests.

High-risk group, low folate levels and low DTI-ALPS index; moderate-risk group, low folate levels and high DTI-ALPS index or high folate levels and low DTI-ALPS index; low-risk group, high folate levels and high DTI-ALPS index. MMSE, Mini-Mental State Examination; MoCA, Montreal Cognitive Assessment. DTI-ALPS, diffusion tensor image analysis along the perivascular space
