# Supplementary material for: Association between circulating biomarkers of one-carbon metabolism and glymphatic system function in cognitive decline of Alzheimer’s disease
Source: Front Neurol. 2026 May 11;17:1779257. doi: 10.3389/fneur.2026.1779257 (PMC13199100; doi:10.3389/fneur.2026.1779257)
Supplement: Supplementary file 7 [file Table_7.docx]

**Table S7.** Correlations between circulating biomarkers of one-carbon metabolism and the DTI-ALPS index in the NC group.

| **Variables** | **Variables** | **Correlation coefficient** | **p value** | **p_FDR_ value** |
| --- | --- | --- | --- | --- |
| Folate | DTI-ALPS index | 0.038 | 0.784^a^ | 0.882^a^ |
|  | Left DTI-ALPS index | -0.005 | 0.972^a^ | 0.972^a^ |
|  | Right DTI-ALPS index | 0.069 | 0.615^a^ | 0.791^a^ |
| Vitamin B12 | DTI-ALPS index | 0.263 | **0.043^a^** | 0.194^a^ |
|  | Left DTI-ALPS index | 0.203 | **0.012^a^** | 0.270^a^ |
|  | Right DTI-ALPS index | 0.286 | 0.027^a^ | 0.194^a^ |
| Homocysteine | DTI-ALPS index | -0.143 | 0.243^b^ | 0.437^b^ |
|  | Left DTI-ALPS index | -0.073 | 0.555^b^ | 0.791^b^ |
|  | Right DTI-ALPS index | -0.195 | 0.111^b^ | 0.270^b^ |

Note:DTI-ALPS, diffusion tensor image analysis along the perivascular space.

^a^ p-value obtained from Pearson correlation analysis. ^b^ p-value obtained from Spearman correlation analysis.
